# Metabolites can regulate stem cell behavior through the STAT3/AKT pathway in a similar trend to that under hypoxic conditions

**DOI:** 10.1038/s41598-019-42669-x

**Published:** 2019-04-16

**Authors:** Gun-Jae Jeong, Donglim Kang, Ae-Kyeong Kim, Kyu-Hyun Han, Hye Ran Jeon, Dong-ik Kim

**Affiliations:** Division of Vascular Surgery, Samsung Medical Center, Sungkyunkwan University School of Medicine, Seoul, Republic of Korea

## Abstract

Stem cell therapy has long been considered a promising mode of treatment for many incurable diseases. Human mesenchymal stem cells (hMSCs) have provided the most promising results to date for regenerative medicine. Nevertheless, due to several obstacles such as difficulty in sourcing and characterizing hMSCs, they remain largely unavailable for clinical use. The signaling requirements for maintaining stem cell function have been studied widely, but little is known about how metabolism contributes to stem cell function. hMSCs have been shown to promote therapeutic efficacy in hypoxic conditions through metabolic conversion. According to published studies, certain metabolites are able to convert stem cell metabolism from oxidative phosphorylation to glycolysis. In this study, we selected several metabolites (fructose-1,6-bisphosphate (FBP), Phosphoenolpyruvic acid (PEP) and sodium oxalate (OXA)) to examine the relation between metabolites and stem cell functions. In addition, we investigated the ability of selected metabolites to induce rapid expansion of this cell population. Our results indicate that selected metabolites stimulate stem cell proliferation by induce glycolytic metabolism via AKT/STAT signaling.

## Introduction

The current excitement regarding the potential for stem cell therapy to improve patient outcomes is understandable. However, several challenges remain with respect to the use of stem cells in scientific, ethical, and political realms. Despite the absence of compelling evidence from adequate, well-controlled clinical trials, human mesenchymal stem cells (hMSCs) may nevertheless be effective therapies for conditions currently considered incurable^1,2^.

Mesenchymal stem cells (MSCs) have been shown to promote therapeutic efficacy via a mechanism that is potentiated by hypoxic conditions (low O_2_)^3^. In particular, hypoxia increases the self-renewal capacity of stem cells through metabolic conversion^4^. According to published studies, certain metabolites are able to convert stem cell metabolism from oxidative phosphorylation to glycolysis. Among these metabolites, fructose-1,6-bisphosphate (FBP), 3-phosphoglyceric acid (3PG), Phosphoenolpyruvic acid (PEP), 2-deoxyglucose (2DG), indoleacetic acid (IAA), and sodium oxalate (OXA) have been shown to have promising effects *in vitro* in several cancer and progenitor cell line models^5–8^. However, systematic identification of effective bioactive agents is challenging, partly due to their low specificity for molecular targets.

Bioactive compounds target glycolytic signaling pathways including protein kinase B (AKT) and signal transducer and activator of transcription (STAT) in addition to other signaling pathways associated with stem cell differentiation and progression. Importantly, natural compounds represent an invaluable resource for synergetic combinatorial treatments^9^. Indeed, the ability of these compounds to target multiple pathways might be advantageous in that they may limit compensatory signaling feedback loops and cross-talk between cellular pathways as well as between different cell types within the stem cell microenvironment.

In the present study, we investigated the molecular effects and relationship between hypoxic condition and metabolites on hMSCs. Our results will improve the understanding of the underlying mechanisms of metabolites on hMSCs and may also expand our understanding of stem cells. Ultimately, our results may increase the feasibility of stem cell therapy.

## Results

### Enhanced cell proliferation in hypoxic conditions through metabolic regulation

**Figure 1:**
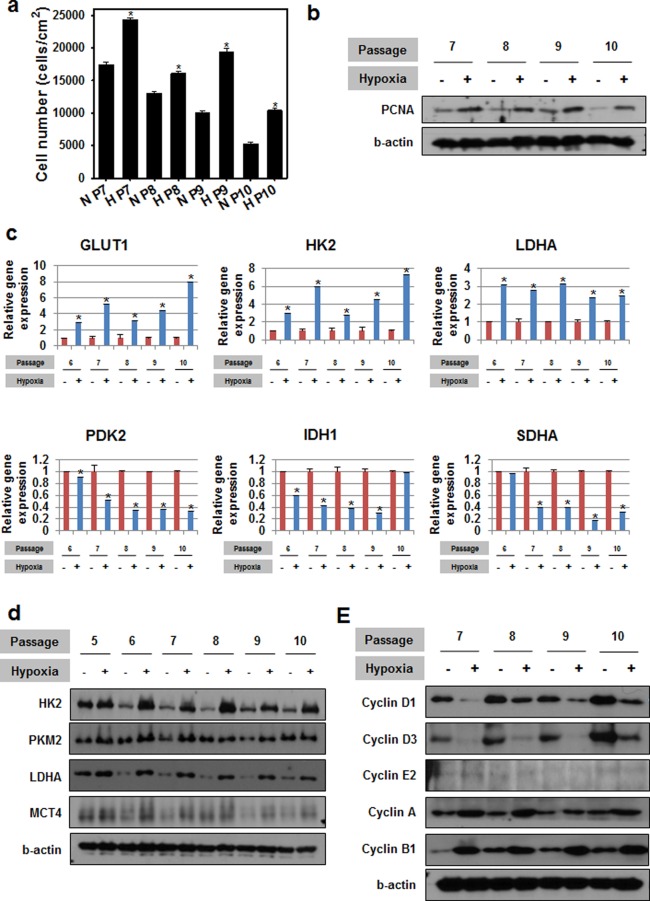
Enhanced cell proliferation under hypoxic conditions is mediated by metabolic regulation. (**a**) Cell numbers after growth under hypoxic or normoxic conditions. *p < 0.05 compared to the normoxia group at each passage; n = 4 per group. (**b**) Proliferating cell nuclear antigen (PCNA) protein expression after culture under hypoxic conditions. (**c**) Expression of genes related to glycolysis (GLUT1, HK2 and LDHA) and oxidative phosphorylation (PDK2, IDH1 and SDHA) after culture under hypoxic conditions. *p < 0.05 compared to the normoxia group at each passage; n = 4 per group. (**d**) Cell metabolism signaling protein expression after hypoxia treatment. (**e**) Expression of cyclin proteins following culture under hypoxic conditions. For all studies, ‘hypoxic conditions’ refers to incubation at 1% O_2_ for 72 hrs.

We first examined the effect of hypoxic conditions on hMSC metabolism. To this end, hMSCs between passages 7 and 10 were cultured for 72 hrs under normoxic (20% oxygen) or hypoxic (1% oxygen) conditions. The number of hMSCs at the end of the culture period was greater under hypoxic conditions compared to normoxic conditions (Fig. 1a). In addition, the protein expression of proliferation cell nuclear antigen (PCNA) in the hypoxic condition group was higher than that of the normoxic group (Fig. 1b). Compared to hMSCs cultured under normoxic conditions, cells cultured under hypoxic conditions exhibited upregulation of glycolysis related genes (glucose transporter 1 (GLUT1), hesokinase2 (HK2), and lactate dehydrogenase A (LDHA)) and downregulation of TCA cycle related genes (pyruvate dehydrogenase kinase isoform 2 (PDK2), isocitrate dehydrogenase 1 (IDH1), and succinate dehydrogenase complex subunit A (SDHA)) (Fig. 1c). In addition, the expression of glycolysis-related proteins (HK2, pyruvate kinase isozymes M2 (PKM2), LDHA, and monocarboxylate transporter 4 (MCT4)) was increased cells cultured under hypoxic conditions compared to cells cultured under normoxic conditions (Fig. 1d). Cells cultured under hypoxic conditions also exhibited upregulation of Cyclin A and B1 and downregulation of Cyclin D1 and D3 protein expression compared to cells cultured under normoxic conditions (Fig. 1e).

### Conversion of hMSC metabolism by selected metabolites

**Figure 2:**
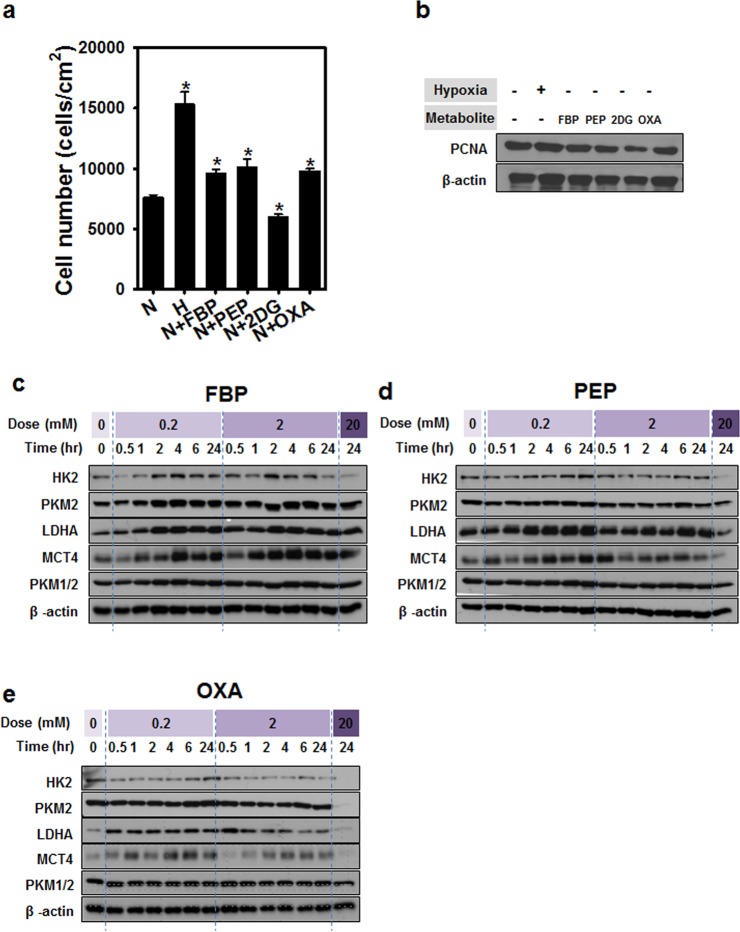
Conversion of hMSC metabolism by selected metabolites. (**a**) Expression of proteins related to cell metabolism after metabolite treatment. Cell numbers (**b**) and expression of proliferating cell nuclear antigen (PCNA) (**c**) after metabolite treatment. (**d**–**f**) Expression of proteins involved in cell metabolism signaling after treatment with the indicated metabolites (FBP, PEP, OXA) according to dose and duration of treatment.

To investigate stem cell metabolism, we treated hMSCs for 72 hrs with different metabolites related to glycolysis, namely, FBP, PEP, 2DG, and OXA, and evaluated their effects on cell proliferation and PCNA expression (Fig. 2a,b). Among the selected metabolites, treatment with hypoxia, FBP, PEP, or OXA resulted in increased cell numbers compared to cells cultured under normoxic conditions, but 2DG treatment showed lower cell numbers compared to that of normoxic conditions. The expression of PCNA protein of 2DG treatment was lower than normoxic condition and other metabolite treatment. In order to examine the relationship between glycolysis and metabolite treatment, hMSCs were also treated with FBP, PEP, or OXA at varying doses and treatment durations. The expression of proteins related to glycolysis, namely, HK2, PKM2, LDHA, and MCT4, were increased following metabolite treatment (Fig. 2c–e).

### Maintenance of hMSC characteristics after metabolite treatment

**Figure 3:**
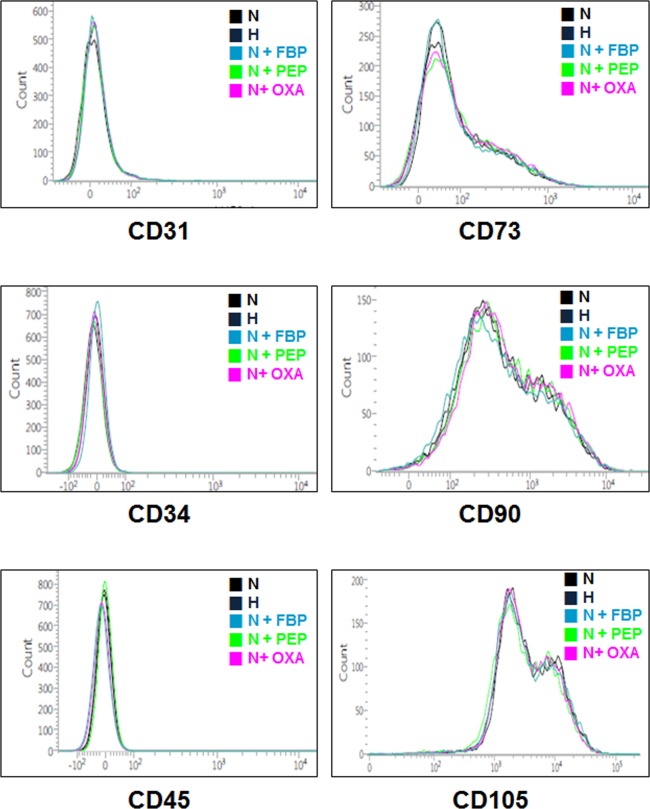
Maintenance of hMSC characteristics after metabolite treatment. Flow cytometry analysis for hMSC markers (CD31, CD73, CD34, CD45 negative and CD 90, CD105 positive) after treatment with the indicated metabolites (FBP, PEP, OXA).

To investigate the characteristics of hMSCs after treatment with metabolites, cells were stained with MSC markers (CD31, CD34, CD45, CD73, CD90, and CD105) and analyzed by FACS. hMSCs treated with the different metabolites exhibited nearly identical characteristics as untreated hMSCs as well as hMSCs cultured under hypoxic conditions (Fig. 3).

### Downregulation of markers of differentiation and senescence after metabolite treatment

**Figure 4:**
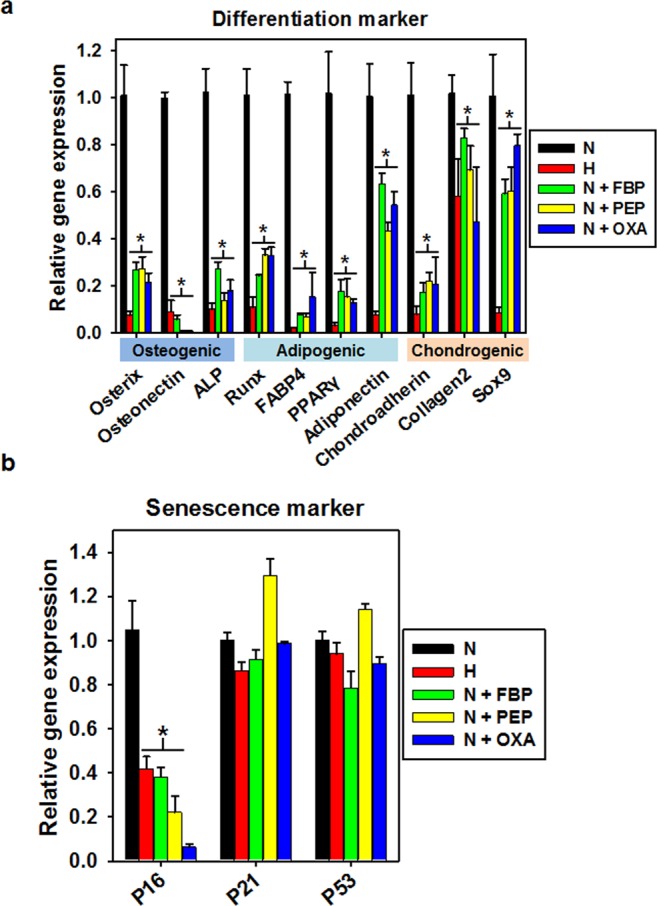
Downregulation of differentiation markers and senescence marker following metabolite treatment. (**a**) Gene expression of markers specific for osteogenic, adipogenic, and chondrogenic lineage differentiation after culturing under hypoxic conditions or with the indicated metabolites (FBP, PEP, or OXA). *p < 0.05 as compared with N group; n = 4 per group. (**b**) Gene expression of markers for senescence after culturing under hypoxic conditions or with the indicated metabolites (FBP, PEP, or OXA). *p < 0.05 as compared with N group; n = 4 per group.

To examine the effect of the different metabolites of hMSC differentiation, we investigated their effect on osteogenic (Osterix, Osteonectin, and alkaline phosphatase (ALP)), adipogenic (runt related transcription factor (Runx), fatty acid binding protein 4 (FABP4), peroxisome proliferator-activated receptor gamma (PPARγ), and Adiponectin), and chondrogenic (Chondroadherin, Collagen2, and Sox9) differentiation markers. hMSCs treated with the metabolites exhibited significantly decreased expression of differentiation markers compared to hMSCs cultured under normoxic conditions. But downregulation effect of differentiation markers after metabolite treatment was smaller than that of hypoxic condition culture (Fig. 4a). We also investigated the effect of metabolite treatment on levels of senescence marker genes. Especially, we found that the gene expression of p16 was decreased in hMSCs treated with metabolites compared to cells cultured under normoxic conditions. On the other hand, there was no significant difference in the gene expression of p21 and p53 between hMSCs cultured with the metabolites or under normoxic conditions (Fig. 4b).

### Cell cycle transition and AKT/STAT pathway change after metabolite treatment

**Figure 5:**
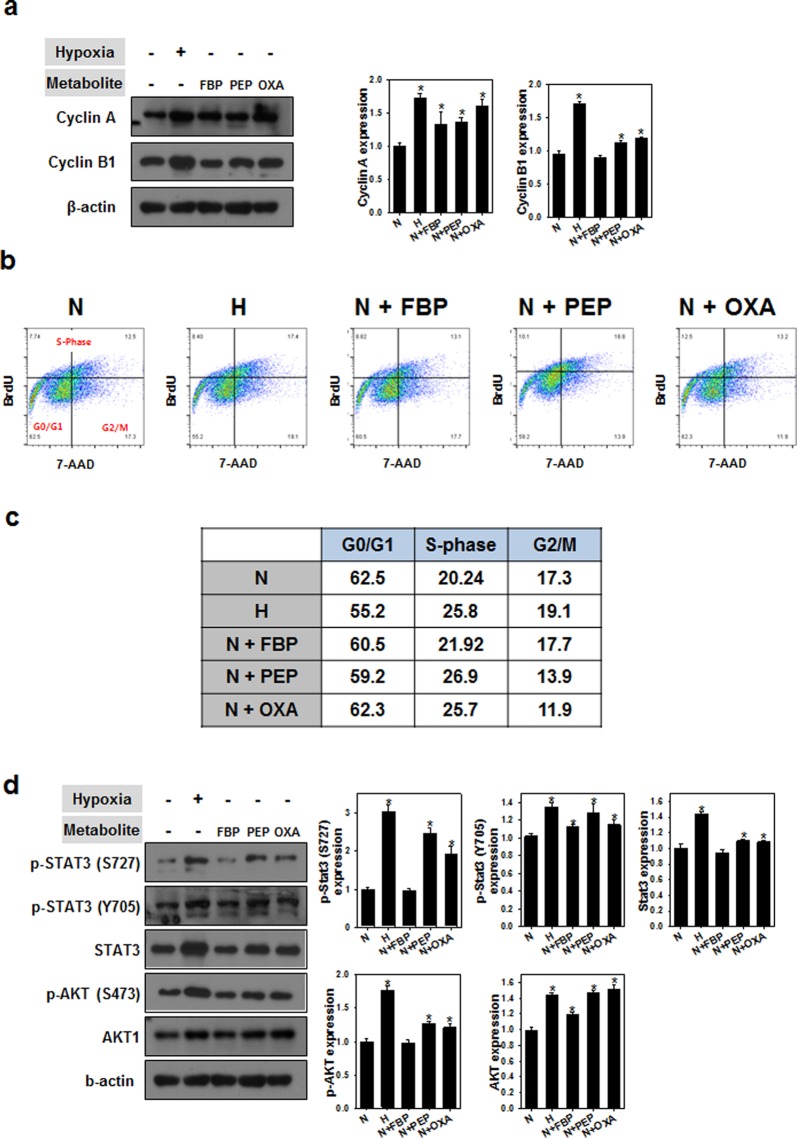
Cell cycle transition after metabolite treatment. (**a**) Cyclin protein expression after culturing under hypoxic conditions or with the indicated metabolites (FBP, PEP, or OXA). *p < 0.05 as compared with N group; n = 4 per group. (**b**,**c**) Cell cycle analysis after culturing under hypoxic conditions or with the indicated metabolites (FBP, PEP, or OXA). (**d**) Western blot analysis and its quantification about expression of AKT/STAT signaling proteins after metabolite treatment. *p < 0.05 as compared with N group; n = 4 per group.

We next examined hMSCs treated with metabolites by cell cycle analysis and western blot assay for proteins involved in cell cycle progression. Among the selected metabolites, cells treated with FBP and OXA exhibited increased expression of cyclin A and B1 (Fig. 5a). Likewise, the percentage of cells in the S phase of growth was higher in hMSCs treated with FBP or OXA compared to cells cultured under normoxic conditions (Fig. 5b,c). The expression levels of AKT/STAT proteins after treatment with the selected metabolites were also increased compared to cells cultured under normoxic conditions (Fig. 5d).

## Discussion

Hypoxic condition is natural microenvironments of the stem cell niches that maintain the MSCs *in vivo*^10,11^. Maintenance of the MSCs under hypoxic condition *in vitro* showed enhanced cell proliferation and increased PCNA protein expression (Fig. 1a,b). The cell cycle related proteins were changed under hypoxic culture (Fig. 1e). This results are in agreement with previous studies cultured MSCs *in vitro* under hypoxic conditions^12–16^. The PCR data indicates hypoxic condition can regulate metabolism of the MSCs through upregulation of glycolysis related genes and downregulation of TCA cycle related genes (Fig. 1c). In addition, expression of glycolysis related proteins were upregulated under hypoxic condition culture (Fig. 1d). This finding suggests that enhanced proliferation of MSCs cultured under hypoxic conditions is related to changed metabolic pathway. Metabolic regulation under hypoxic culture in stem cells was studied previously^4,17,18^, but correlation of changed metabolic pathway through treatment of metabolites and the MSCs behavior has not been revealed perfectly.

Metabolism plays a pivotal role in controlling whether a cell will proliferate, differentiate, or remain quiescent. A key unresolved question in this paradigm is how metabolism integrates with epigenetic and genetic programs to regulate coordinately stem cell function and fate^17,19–26^. Numerous studies have shown that mouse and human ESCs and iPSCs, or highly pluripotent stem cells, have an increased dependence on glycolysis under aerobic conditions compared to cells utilizing oxidative phosphorylation to meet their energy needs. The molecular mechanisms that regulate both energy metabolism in pluripotent stem cells and the changes that occur during differentiation or reprogramming remain an area of active scientific study^27,28^. For example, glycolysis regulating enzymes including hexokinase and lactate dehydrogenase A are highly expressed in pluripotent stem cells^21^. Consistent with previous studies, we treated four metabolites to the MSC and found that glycolysis metabolism was induced by certain concentration of FBP, PEP and OXA (Fig. 2), all of which play roles in stem cell metabolic conversion and proliferation^5–8^. Treatment of FBP, PEP and OXA did not induced MSC characterization change compared with normoxic or hypoxic culture (Fig. 3), but caused decrease of differentiation markers and senescence markers (Fig. 4). Decrease of senescence markers may affect to paracrine functions and differentiation markers expression of MSC^15,29,30^. In addition, metabolites treatment induced cyclin A and cyclin B1 expression (Fig. 5a) and cell cycle change (Fig. 5b,c). Especially in PEP and OXA treated group showed increased protein expression of STAT3 and AKT pathway (Fig. 5d). It is well known that AKT/STAT pathway can regulate cell cycle^31–33^. The tendency of protein expression and gene expression of metabolite treated MSCs were similar with that of the cells cultured under hypoxic condition, but the change of protein and gene expression was smaller than that of the hypoxic condition culture. Our results suggested that treatment with certain metabolites is an effective means of enhancing MSCs maintenance, which is important with regard to the use of these cells in regenerative medicine, without hypoxic condition.

The results of the present study have the potential to have a significant impact on the field of stem cell culture and, more broadly, the study of diseases that may benefit from adult stem cell implantation. Therefore, our study not only highlights the importance of metabolism maintaining stem cells, but suggests that understanding the metabolic changes associated with stem cell may shed light on the metabolic mechanisms that regulate cellular proliferation. On the other hand, the results of the present study, while important, are not sufficient to generate a complete picture of the molecular components regulating stem cell function and differentiation. As metabolism involved in every aspect of cell function, future studies may focus on understanding the effect of metabolism on cell fate.

## Materials and Methods

### Cell culture

Human umbilical cord derived mesenchymal stem cells (hMSCs; PromoCell) were grown in Dulbecco’s modified Eagle’s medium (DMEM; GIBCO) containing 10% fetal bovine serum (GIBCO) and 1% penicillin/streptomycin antibiotics (Invitrogen) at 37 °C in a 5% CO_2_ incubator with either 21% O_2_ (normoxia) or 1% O_2_ (hypoxia). The hypoxic condition culture cells were maintained in hypoxia after passage number 3. For cell number counting, cells were stained with Trypan blue (Invitrogen) and cell densities were determined with a Countess (Thermo Fisher Scientific) automated cell counter. The initial density of seeded cell 5000 cells per cm^2^. Cell number counting experiments were performed at 6 day after seeding for Fig. 1a and 3 day after seeding for Fig. 2a. Passage number 8 cells were used for metabolite treatment experiment.

### Western blotting

Cells were lysed in LIPA buffer and the lysates were clarified by centrifugation at 13,200 rpm for 30 min at 4 °C. Protein lysates were subjected to SDS-PAGE and transferred to membranes. Membranes were blocked in TBS-T (20 mM Tris, 137 mM NaCl, 0.1% Tween-20, pH7.4) containing 5% skim milk and then incubated with the primary antibody at an appropriate dilution in TBS-T containing 5% skim milk for 3 h at room temperature or overnight in a cold room. Staining with a secondary antibody (anti-rabbit- or anti-mouse-conjugated horseradish peroxidase; Cell signaling and Bethyl, respectively) was followed by ECL (AB Frontier) for visualization.

### RNA isolation and quantitative real-time PCR

Total RNA was extracted using and RNeasy Mini Kit (Qiagen) and quantified using a Nanodrop 2000 spectrophotometer (Thermo Fisher Scientific). cDNA was prepared with a PrimeScriptTM RT reagent kit (Takara) using Oligo d(T) and Random primers for qRT-PCR. Primers for the following genes were used for our analysis: GLUT1, HK2, LDHA, PDK2, IDH1, SDHA, p53, p21, p16, Osterix, Osteonectin, ALP, Runx, FABP4, PPARγ, Adiponectin, Chondroadherin, Collagen2 and Sox9.

### Metabolite treatment

Fructose-1,6-Bisphosphate (FBP), phosphoenolpyruvic acid (PEP), 2-Deoxyglucose (2DG), and Sodium oxalate (OXA) were purchased from Sigma Aldrich. Cells were treated with FBP or PEP at a concentration of 200 μM or with 2DG or OXA at a concentration of 100 μM for 72 hrs.

### Flow cytometry analysis

Human UC-MSCs were evaluated using surface marker detection at passage 8 (P 8) to confirm the effect of metabolite treatment on MSC characterization. hUCB-MSCs at 80% confluence were harvested and suspended in FACS buffer (1 × 10^7^ cells/ml). The following antibodies were then added to each sample: Anti-CD34 fluorescein isothiocyanate (FITC), Anti-CD90 phycoerythrin (PE), Anti-CD73-PerCP-Cy5.5, Anti- CD105-allophycocyanin (APC), Anti-CD31 Violet450 (V450), and Anti-CD45 Violet500 (V500) (BD Biosciences) followed by incubation at 4 °C for 30 min. For cell cycle analysis, hUCB-MSCs treated with hypoxic condition and metabolites were stained with Phase-Flow BrdU Cell Proliferation Kit (BioLegend). After staining, cells were washing with PBS and evaluated with a BD FACS Verse flow cytometer (BD Biosciences). Data were analyzed with BD Verse analysis software (BD Biosciences).

### Statistical analysis

Quantitative data were expressed as mean ± SD. OriginPro 8 software (OriginLab, Northampton, MA) served for one-way analysis of variance. “Statistically significant” means that the *p* value is less than 0.05.

## Supplementary information

supplementary info
