# Supplementary material for: Metabolites can regulate stem cell behavior through the STAT3/AKT pathway in a similar trend to that under hypoxic conditions
Source: Sci Rep. 2019 Apr 16;9:6112. doi: 10.1038/s41598-019-42669-x (PMC6468014; doi:10.1038/s41598-019-42669-x)

(Supplementary information)

Metabolites can regulate stem cell behavior through the STAT3 / AKT pathway in a similar trend to that under hypoxic conditions

Gun-Jae Jeong‡, Donglim Kang‡, Ae-Kyeong Kim, Kyu-Hyun Han, Hye Ran Jeon, Dong-ik Kim*.

Division of Vascular Surgery, Samsung Medical Center, Sungkyunkwan University School of Medicine, Republic of Korea

‡ Authors whom contributed equally this work.

***Author to whom the correspondence should be addressed:**
Dong-ik Kim, M.D., Ph.D. Division of Vascular Surgery, Samsung Medical Center, 81 Irwonro, Gangnam-gu, Seoul 06351, Republic of Korea. TEL: +82-2-3410-3467, FAX: +82-2-

3410-0040,

E-mail: dikim@skku.edu (D.-I. Kim).

Supplementary Figure 1. Original developed image for western blot analysis. (A) For figure 1. B and (B), (C) for figure 1. D.

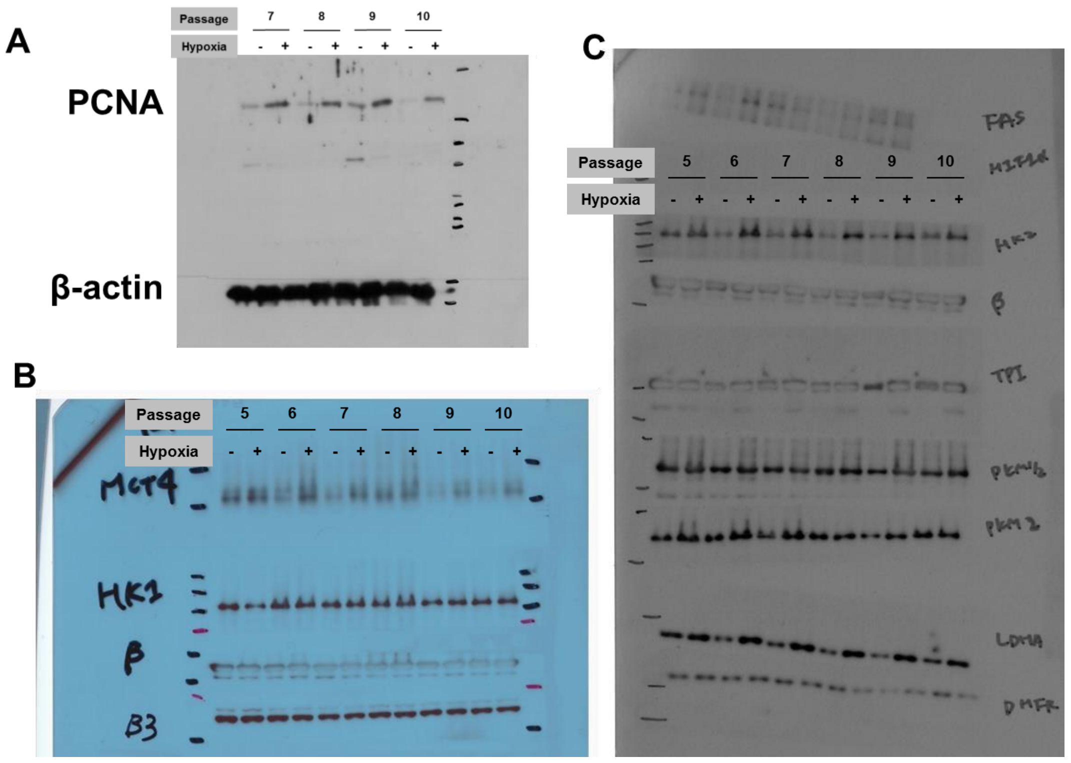

Supplementary Figure 2. Original developed image for western blot analysis. (A) For figure 2. C and D. (B) For figure 2. E.

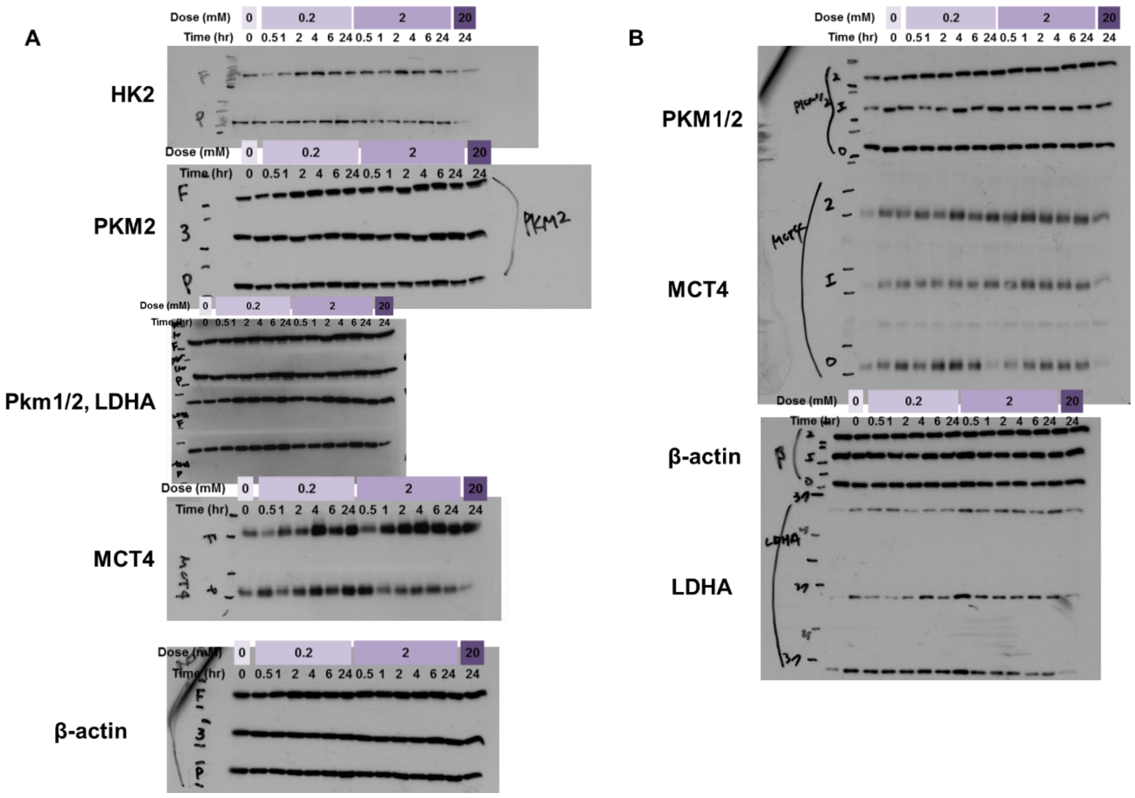

Supplementary Figure 3. Original developed image for western blot analysis for Figure 5.

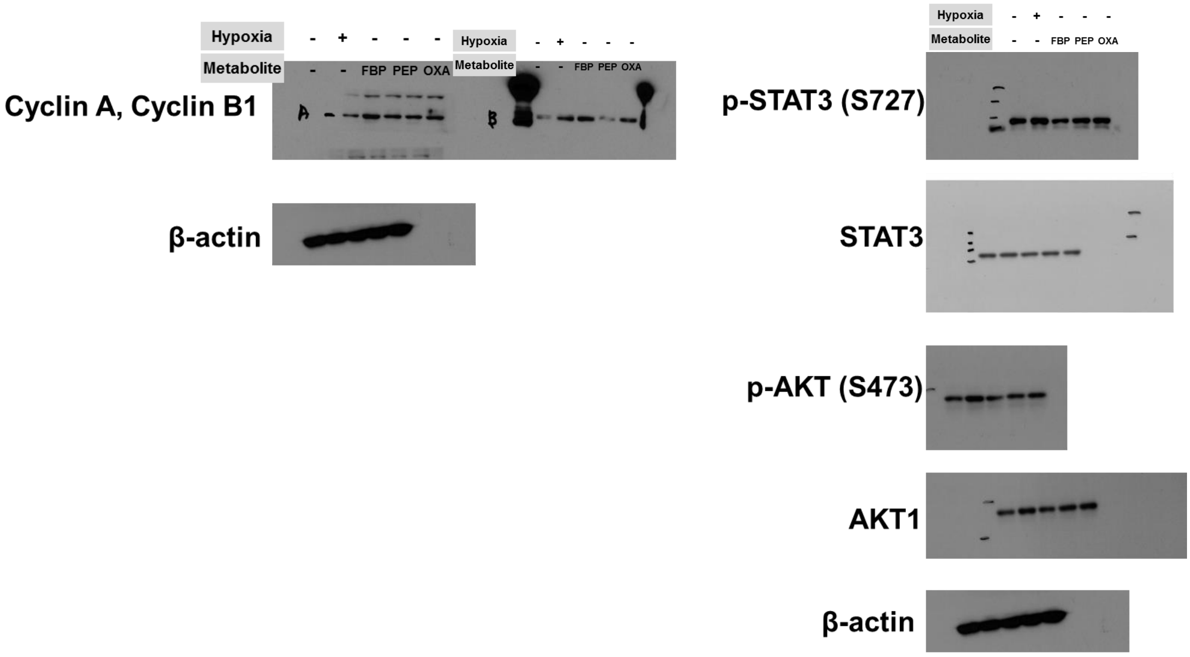

Supplement: Supplementary file 1 — supplementary info [file 41598_2019_42669_MOESM1_ESM.pdf]
